# *CHL1* suppresses tumor growth and metastasis in nasopharyngeal carcinoma by repressing PI3K/AKT signaling pathway via interaction with Integrin β1 and Merlin

**DOI:** 10.7150/ijbs.34785

**Published:** 2019-07-11

**Authors:** Juan Chen, Chen Jiang, Li Fu, Cai-Lei Zhu, Yan-Qun Xiang, Ling-Xi Jiang, Qian Chen, Wai Man Liu, Jin-Na Chen, Li-Yi Zhang, Ming Liu, Chao Chen, Hong Tang, Bo Wang, Sai Wah Tsao, Dora Lai-Wan Kwong, Xin-Yuan Guan

**Affiliations:** 1 Departments of Clinical Oncology, Li Ka Shing Faculty of Medicine, The University of Hong Kong, Hong Kong, China;; 2 Department of Clinical Oncology, The Seventh Affiliated Hospital, Sun Yat-sen University.; 3 Departments of Pathology, Li Ka Shing Faculty of Medicine, The University of Hong Kong, Hong Kong, China;; 4 Departments of Anatomy, Li Ka Shing Faculty of Medicine, The University of Hong Kong, Hong Kong, China;; 5 State Key Laboratory of Oncology in Southern China, Sun Yat-Sen University Cancer Center, Guangzhou, China;; 6 Department of Nasopharyngeal, Sun Yat-Sen Cancer Center, Guangzhou, China.; 7 Guangdong Key Laboratory for Genome Stability & Disease Prevention, Department of Pharmacology and Shenzhen University International Cancer Research Centre, Shenzhen University school of Medicine, Shenzhen, China.; 8 Department of Orthopedics, Union Hospital, Tongji Medical College, Science and Technology of Huazhong University, Wuhan, China.

**Keywords:** nasopharyngeal carcinoma, tumor suppressor gene, tumor invasion, tumor metastasis

## Abstract

Deletion of Chromosome 3p is one of the most frequently detected genetic alterations in nasopharyngeal carcinoma (NPC). We reported the role of a novel 3p26.3 tumor suppressor gene (TSG) *CHL1* in NPC. Down-regulation of *CHL1* was detected in 4/6 of NPC cell lines and 71/95 (74.7%) in clinical tissues. Ectopic expressions of CHL1 in NPC cells significantly inhibit colony formation and cell motility in functional study. By up-regulating epithelial markers and down-regulating mesenchymal markers *CHL1* could induce mesenchymal-epithelial transition (MET), a key step in preventing tumor invasion and metastasis. *CHL1* could also cause the inactivation of RhoA/Rac1/Cdc42 signaling pathway and inhibit the formation of stress fiber, lamellipodia, and filopodia. *CHL1* could co-localize with adhesion molecule Integrin-β1, the expression of *CHL1* was positively correlated with Integrin-β1 and another known tumor suppressor gene (TSG) Merlin. Down-regulation of Integrin-β1 or Merlin was significantly correlated with the poor survival rate of NPC patients. Further mechanistic studies showed that *CHL1* could directly interact with integrin-β1 and link to Merlin, leading to the inactivation of integrin β1-AKT pathway. In conclusion, *CHL1* is a vital tumor suppressor in the carcinogenesis of NPC.

## Introduction

Nasopharyngeal carcinoma (NPC) is a widespread head and neck malignancy that poses a serious threat to health especially in Southeast Asia 1. Although the molecular mechanism underlying the development of NPC remains unclear, some factors such as genetic susceptibility, EBV infection and environmental risk have been associated with its pathogenesis 2, 3. Cytogenetic studies have been able detect multiple recurrent chromosomal abnormalities in NPC including the deletion of the short arm of chromosome 3p, suggesting that the resident genes at this region play important roles in NPC pathogenesis 4-6. Studies has showed that the loss of chromosome 3p was recurrently observed in several carcinogenic disease including NPC 7, lung cancer 8 and esophageal carcinomas 9 . By using single-nucleotide polymorphism (SNP)-mass array, we identified the corresponding tumor suppressor genes (TSGs) commonly deleted at 3p in non-small cell lung cancer 10 and esophageal squamous cell cancer 11. A putative TSG called cell close homologue of L1 (*CHL1*, also known as *CALL*) located at 3p26 loci was identified because it is commonly deleted in lung, esophageal and nasopharyngeal carcinoma respectively.

*CHL1* encodes a protein with 1,224 amino acids belonging to a member of the L1 gene family of neural cell adhesion molecules (CAM) 12. CHL1 protein contains a single-pass transmembrane peptide, 6 immunoglobulin (Ig) domains, 4 fibronectin type III (FNIII) domains, and a 105-amino acid cytoplasmic domain 13. Being a neurotransmitter, *CHL1* has been reported to be involved in the general cognitive activities 14, 15. It is also reported to be involved in some neurological diseases such as schizophrenia 16. Deleting a copy of this gene may be associated with mental dysfunction of 3p- syndrome 14. As a CAM member, *CHL1* and *L1CAM* another CAM have been reported in cancer development and metastasis in ovarian 17, breast 18, colon 19, pancreatic 20 and gallbladder 21 cancers. A study has also reported that 3p26 is a major predisposition locus for prostate cancer 22. Collectively these findings suggest that *CHL1* is involved in the carcinogenesis. In this study, the expression status of *CHL1,* its potential tumor suppressive role and molecular mechanism in NPC development were characterized.

## Materials and Methods

### Cell lines and clinical samples

Two immortalized nasopharyngeal epithelial cell lines (NP460 and NP69) and three NPC cell lines (C666, CNE2 and SUNE1) were used in this study. C666 was derived from EBV-positive NPC, while SUNE1 and CNE2 from EBV-negative NPC cell line. Both NP69 and NP460 are EBV-negative cell lines. NPC cell lines were cultured in high-glucose Dulbecco's Modified Eagle Medium (DMEM) (Gibco BRL, Grand Island, NY) supplemented with 10% fetal bovine serum (FBS). NP460 and NP69 on the other hand were cultured in a defined keratinocyte serum-free medium (Invitrogen, Carlsbad, CA). Guangzhou Cohort consisting of 95 NPC tumor biopsies were obtained from Sun Yat-Sen University Cancer Center (Guangzhou, China). The clinical samples used in this study were reviewed and approved by the Committee for Ethical Review of Research involving Human Subjects at Sun Yat-sen University. Hong Kong Cohort consisting of 15 tumor tissues of primary nasopharyngeal carcinoma and their matched non-tumor tissues endoscopic biopsies were taken from the Queen Mary Hospital in Hong Kong. The samples in this study were based on the standard method approved by the Ethics Review Committee of research involving Human Subjects at the University of Hong Kong.

### Quantitative Real-time PCR (qPCR)

Quantitative Real-time qPCR was performed using a SYBR Green PCR kit (Applied Biosystems, Foster City, CA) following the instructional protocol contained in manufacturer's manual and the signal detection was obtained using ABI PRISM7900 Sequence Detector (Applied Biosystems, Foster City, CA). GAPDH was an internal control. The data were collected and analyzed with SDS Relative Quantification Software 2.2.3 (Applied Biosystems, Foster City, CA).

### Promoter methylation analysis

CpG islands were predicted using the MethPrimer software. We extracted Genomic DNA from cell lines with bisulfite treatment method using Epitect Bisulfite Modification Kit (Qiagen, Valencia, CA). Bisulfite genomic sequencing (BGS) was performed with *CHL1* primers (Forward primer: 5'-TTTTAAATGAAGGAAAGTAAGAAG-3'; Reverse primer: 5'-TCTACTCCCTTCCTAAATTCTAC-3'). The reagent 5-aza-2′-deoxygcitidine (Sigma-Aldrich, St Louis, MO) was used to test if *CHL1* expression could be restored by demethylation treatment, it was also employed for the extraction of total RNA (qPCR method) which we used to detect the expression of *CHL1*.

### *In vitro* tumorigenicity assays

Tumor suppressive function of *CHLI* was studied by cloning a full-length *CHL1* into pcDNA3.1 vector (Invitrogen, Carlsbad, CA) the clones were gently transfected into NPC cell lines C666 (*CHL1*-C666) and SUNE1 (*CHL1*-SUNE1). Non-transfected cells (Vec-C666 and Vec-SUNE1) were used as controls. To measure the growth rate, the cells were implanted into a 96-well plate at a density of 1×10^3^ per well and cultured for 6 days. Cell growth rate was determined using cell proliferation XTT kit (Sigma-Aldrich, St. Louis, MO) at specific time intervals according to manufacturer's instruction. To assay the foci formation, we implanted cells in a 6-well plate at a density of 1×10^3^ cells per well and incubated at 37 ºC for 14 days. Surviving colonies (>50 cells per colony) were stained with 0.1% crystal violet and counted. This experiment was performed independently three times.

### *In vivo* tumorigenicity assay

Approximately 1×10^6^ *CHL1*- and Non-transfected cells, respectively were subcutaneously injected into the bilateral dorsal region of 4-week-old nude mice (n=10 for each group). Tumor growth rate was determined by measuring tumor volume throughout a period of 4-weeks (Formula: Volume= 0.5×L×W^2^). Experiments on animals were carried out according to the protocols approved by the Committee on the Use of Live Animals in Teaching and Research (CULATRA).

### Cell motility assays

For wound healing assay, cells were implanted into a 6-well plate until they are confluent. Cultured cells were wounded with a sterile tip and photographed under a phase contrast microscope at different time intervals. For cell invasion assay, the cells were implanted into a 24-well Biocoat Matrgel Invasion Chamber (BD Biosciences, San Jose, CA) according to the instructional procedures in manufacturers' guide. After 24 hours, cells that invaded through the Matrigel were fixed on a slide using 100% methanol, stained with 0.1% crystal violent and counted under a microscope. This experiment was performed independently three times.

### Experimental metastasis assay

Five-week-old severe combined immunodeficient (SCID)-Beige mice were used for *in vivo* metastasis assay. Briefly, 5×10^5^ cells were injected into the tail vein of each SCID mouse (n=5 for each group). All tested mice were sacrificed at week 12. To determine the number of tumor nodules formed on the surface of the lung sections of tissues from the lungs were extracted and then embedded in paraffin, removed and were stained with H&E. All procedures were approved by the University of Hong Kong CULATR.

### Cell cycle analysis with flow cytometry

*CHL1*- and non-transfected cells (5×10^6^) were cultivated in DMEM medium that is supplemented with 10% FBS. The addition of serum to culture medium was discontinued when 70% of the cells were synchronized with cells at G1 phase. After 72hr of incubation, 10% FBS was added to the medium and incubated for further 12hr. Thereafter the cells were fixed with 70% ethanol, stained with propidium iodide, and DNA content was determined using Cytomics FC (Beckman Coulter, Brea, CA). The results obtained were evaluated using Cell Quest and ModFit LT2.0 software (BD Biosciences, San Jose, CA). Triplicate independent experiments were also performed.

### RNA interference with siRNA

Silencing of *CHL1* expression was achieved using siRNA from Sigma (Sigma-Aldrich, St. Louis, MO). Scramble siRNA obtained from Ambion's predesigned siRNA database (Ambion, Inc., Austin, TX) was used as negative control. siRNA was transiently transfected into NP 460 cells by liposome TM-2000 (Invitrogen, Carlsbad, CA). 48 hours after transfection, the efficiency of gene silencing was analyzed with RT-PCR.

### F-actin staining

The slides attached with cells were fixed with 4% paraformaldehyde in PBS for 10min, followed by staining with 6.6µM Rhodamine phalloidin (Invitrogen, Carlsbad, CA) at room temperature. cells were further counterstained with DAPI and images were collected by a *Leica DMRA* fluorescence microscope (Rueil-Malmaison, Germany).

### Rho-GTPase activation assay

Rhotekin-agarose (for isolating Rho-GTP) and PAK1 PBD-agarose (for isolating Rac1-GTP and Cdc42-GTP) (Upstate Biotechnology, Lake Placid, NY) were used to assay for Rho-GTPase activation following the instructional procedure from manufacturer's guide. The quantification and characterization of Rho, Rac1, and Cdc42 were carried out using western blot assay.

### Western blotting and antibodies

Western blot analysis was performed using the standard method with β-actin as the loading control. Antibodies used in this study are listed in the Supplementary Table 2.

### Immunofluorescence (IF)

The cells transfected with *CHL1* and vectors were placed on gelatin coated tablets, fixed with 4% paraformaldehyde, permeabilized in PBS with 0.1% Triton-X 100, and blocked with 1% bovine serum albumin. The cells were incubated overnight with primary antibodies at 4°C followed by another incubation at room temperature with FITC-conjugated anti-mouse secondary antibody and Texas red-conjugated anti-rabbit secondary antibody for 1 hour. Images were taken after adding anti-fade 4′, 6-diamidino-2-phenylindole (DAPI).

### Co-immunoprecipitation (Co-IP)

Co-immunoprecipitation (Co-IP) assay was performed with immunoprecipitation kit (Roche, Mannheim, Germany) according to the manufacturer's instructions. Briefly after cell lysis, sample preparation, preclearing with protein A/G agarose, and immune-precipitation of the target protein, gel electrophoresis was performed followed by western blotting.

### Statistical analysis

Statistical analysis was carried out using the standard version of SPSS 13.0. Results were presented as mean ±SD. χ^2^ test (categorial variables) or Student t-test (continuous variables) was used to compare data from the two groups. The correlations between Integrinβ1 and CHL1 in clinical samples were analyzed using Pearson correlation. The survival data was analyzed by Kaplan-Meier method and the *P* (Pearson co-efficient) value was calculated with log-rank analysis. A significant difference was considered when *P* value was less than 0.05.

## Results

### *CHL1* is frequently down-regulated in NPC

**Figure 1:**
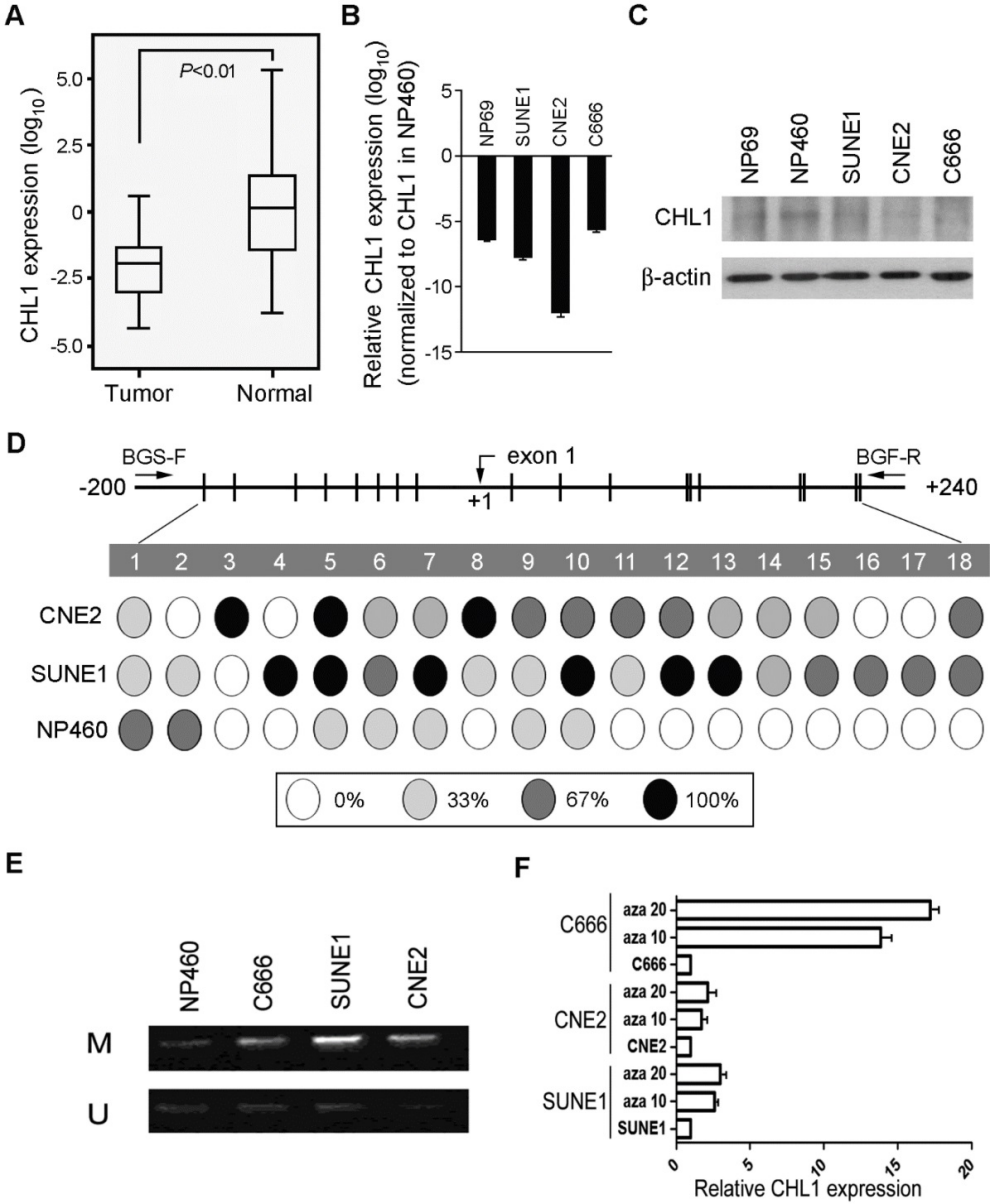
** Downregulation of *CHL1* in NPC**. (A) Quantification and characterization of mRNA levels in *CHL1* of 95 primary NPC cases was obtained using qRT-PCR these results were contrasted with those obtained for normal tissue (N);. *GAPDH* was used as an internal control. (B) *CHL1* Fold changes detected using qRT-PCR in 15 primary NPC tissues and their corresponding non-tumor tissues (N). *CHL1* was normalized by internal control GAPDH (*P<0.01, independent Student* t* test). (C) qRT-PCR analysis of *CHL1* expression in three NPC cell lines (C666, CNE2 and SUNE1) and NP69. The fold change expressions of *CHLI* detected were compared against those of the immortalized NP cell line, NP460. (D) The expression of *CHL1* in the three NPC cell lines, NP 460 and NP69 were characterized by Western blot assay. (E) Mapping of the methylation status of CpG dinucleotides within the *CHL1* promoter region was done with BGS in NPC cell lines (CNE2 and SUNE1) and normal cell line (NP460). A 400-bp region spanning through the CpG island with 18 CpG sites was analyzed. The methylation status at each CpG dinucleotide was represented in the pie charts. The chart shows the methylation activities at three levels indicated with these colours, black, white and grey circle representing completely methylated, completely unmethylated and partially methylated CpGs respectively. (F) Promoter methylation analysis of *CHL1* in NPC cell lines was carried out by MSP, with Immortalized nasopharyngeal epithelial cell line NP460 as normal control. Expressed as M & U; M, methylated allele and U, unmethylated allele respectively. (G) Expression of *CHLI* by qPCR analysis after treatment with 5-aza-dC a demethylation agent in NPC cell lines (C666, CNE2 and SUNE1 cells). Again, GAPDH served as the internal control. Three representing NPC cell lines (C666, CNE2 and SUNE1) without treatment; Aza 10, cell lines treated with 10 µM 5-aza-2'-deoxycytidine; Aza 20, cell lines treated with 20 µM 5-aza-2'-deoxycytidine.

The expression pattern of *CHL1* was analyzed with qPCR using samples obtained from 95 primary NPC patients (patient that has not received any form of treatment prior to the collection of the sample) and compared against the control (pool RNA from 10 non-tumor nasopharyngeal tissues), down-regulation of *CHL1* was detected in 71/95 (74.7%) of NPC cases. (Supplementary Fig. 1A). Kaplan-Meier analysis was used analyze the down-regulation of *CHL1,* the result showed that *CHL1* was not significantly associated with the Disease-Free Survival (DFS) time (P>0.05), however, after 30 months of observation a different result was obtained, as shown in the diagram. (Supplementary Fig 1B). Clinical correlation study also showed that the down-regulation of *CHL1* was not significantly associated with any tested clinical features (Supplementary Table 1). In the paired tumor and non-tumor tissue, down-regulation of *CHL1* was detected in 12/15 (80.0%) of the NPC cases (Fig. 1A). The chart showed a significant difference (*P*<0.01) in the levels of *CHL1* between NPC and paired non-tumor tissues. We also investigated the mechanism of expression of *CHL1* using qPCR and western blot analysis (Fig. 1B & 1C) in 3 NPC cell lines (C666, SUNE1 and CNE2) and 2 immortalized nasopharyngeal (NP) cell lines (NP69 and NP460). The result revealed that down-regulation of *CHL1* was observed in 4 cell lines (NP69, C666, SUNE1 and CNE2), except NP460. To explore the mechanism behind *CHL1* down-regulation, promoter methylation status of *CHL1* in NP460, SUNE1 and CNE2 were characterized by BGS. The results showed that promoter hypermethylation was observed in cell lines of SUNE1 and CNE2 with low expression of *CHL1*, except in NP460 cells where the expression of *CHL1* is high (Fig. 1D). To determine whether the down-regulation of *CHL1* in NPC was caused by hypermethylation in this promoter region, the methylation status of *CHL1* was examined using MSP methylation or unmethylation specific primers in three NPC cell lines (SUNE1, CNE2 and C666), on observation both methylated and unmethylated alleles were expressed in SUNE1 and CNE2 cell lines, except in NP460 where a weak expression of methylated allele is observed (Fig. 1E). In other to confirm the effect of promoter methylation on *CHL1* expression, SUNE1, CNE2 and C666 cells were treated with the demethylating agent 5-aza-2'-deoxycytidine. After the treatment, the expression of *CHL1* was restored (Fig. 1F), which strongly indicated that the promoter hypermethylation was the major vehicle in the mechanism of *CHLI* down-regulation.

### *CHL1* shows strong tumor suppressive ability

**Figure 2:**
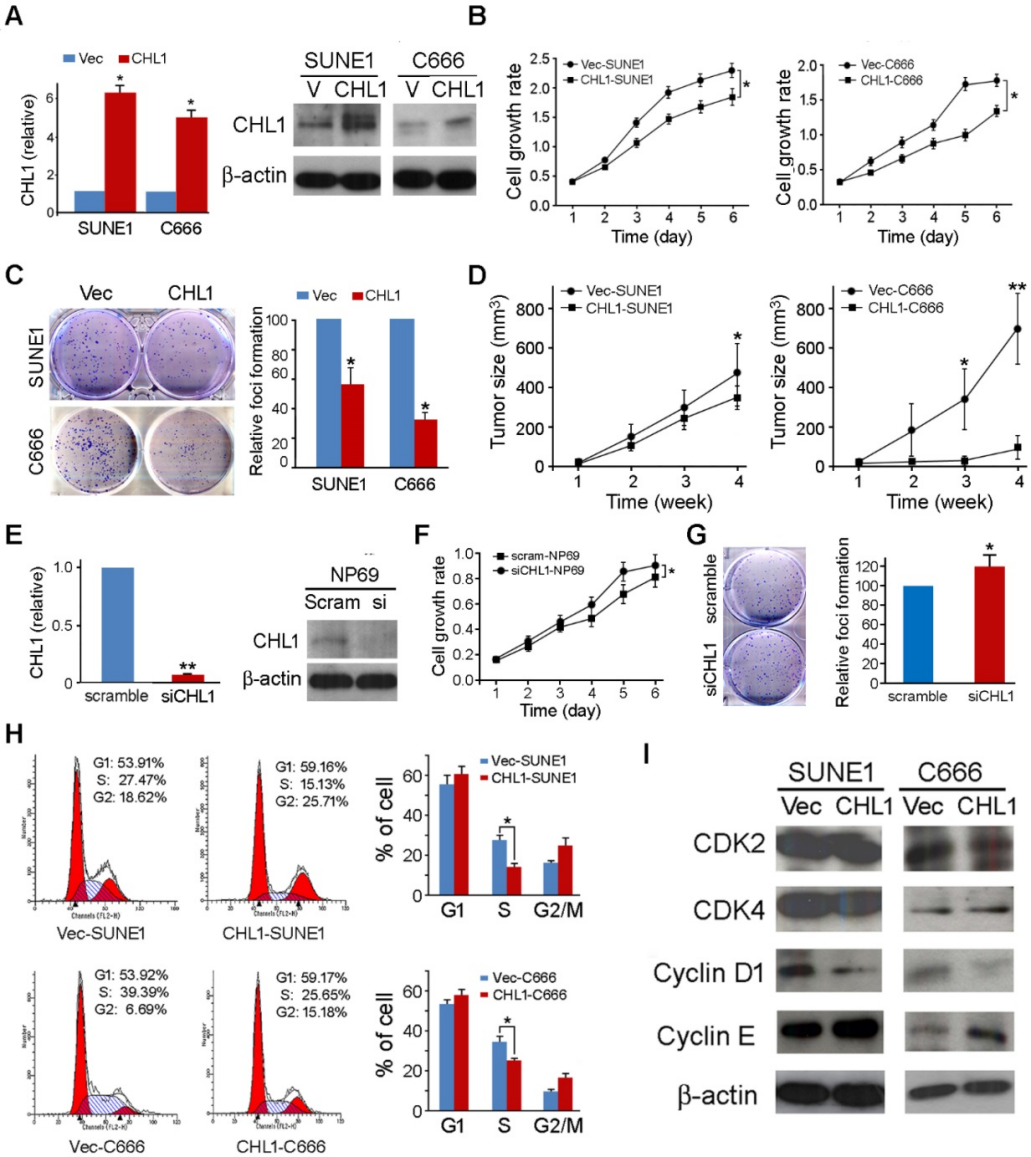
** Tumor suppressive function of *CHL1* in NPC cells.** (A) Ectopic expression of *CHL1* was detected in CHL1-transfected cells by qRT-PCR and western blotting. (B) Cell growth rates of *CHL1*- and non-transfected cells were compared by MTT assay. The results are expressed as mean ±SD of three independent experiments: *P<0.05, **P<0.01, independent Student *t* test. (C) A brief of the decreased foci formation in monolayer culture induced by *CHL1*. Quantitative analyses of foci numbers are indicated in the right panel. Values are displayed as the mean ±SD of at least three independent experiments: *P<0.05, **P<0.01, independent Student *t* test. (D) A brief of tumor formation in nude mice. Tumors induced by vector (left) and clone (right) are indicated by arrows. Excised tumors were shown in the bottom, along with the summary of tumor growth rates in nude mice induced by *CHL1*- and empty vector-transfected NPC cells. The average tumor volume is expressed as mean ± SD in 10 inoculated sites for each group (* *P*<0.05; ** *P*<0.001, Student's t-test). (E) qRT-PCR and Western blot analysis results for the Expression of *CHL1* in si*CHL1* and scramble NP69 cell (** p<0.001, Student's t-test). (F) Comparison of cell growth rates of si*CHL1* and scramble NP69 cells by MTT assay. The results are expressed as mean ±SD of three independent experiments: *P<0.05, **P<0.01, independent Student *t* test. (G) Summary of the foci formation of the scramble cells. Quantitative analyses of foci numbers are shown in the right panel. The values presented as mean ±SD of at least three independent experiments: **P<0.01, independent Student *t* test. (H) Briefs of DNA content detected by flow cytometry showed that the percentage of cells in the S phase was lower while the percentage of cells in the G1 phase was higher in *CHL1*-transfected cells than in non-transfected cells (*p<0.05, Student's t-test). The values are expressed as mean ± SD of three independent experiments. (I) Protein expressions of CDK2, CDK4, cyclin D1 and cyclin E were compared with CHL1- and non-transfected NPC cells. β-actin was used as a loading control.

In other to investigate the anti-tumor ability of *CHL1*, *CHL1* was transfected into 2 nasopharyngeal carcinoma cells (*CHL1*-SUNE1 and *CHL1*-C666) for functional study. Non-transfected cells (Vec-SUNE1 and Vec-C666) were used as controls. Expressions of *CHL1* in *CHL1*- and non-transfected cells were determined using qPCR and Western blot analysis (Fig. 2A). The study of the tumor suppressive role of *CHL1* was characterized by an *in vitro* and *in vivo* assay. The results of the assay portrayed that growth rates were significantly decreased in *CHL1*-transfected cells (*P*<0.01, Student's t-test) compared to the control (Fig. 2B). Foci formation assay showed that *CHL1* have a significant inhibitory effect on *CHL1*-transfected cells (*P*<0.01, Student's t-test) compared to control cells (Fig. 2C).

Moreover, we further investigated the tumor suppressive potential of *CHL1* by *in vivo* tumor formation assay in nude mice. Here we subcutaneously injected *CHL1*- and non-transfected cells into the bilateral dorsal region of nude mice. The Xenograft tumor growth curve showed that tumors induced by *CHL1*-transfected cells grew much slower than tumors induced by non-transfected cells (Fig. 2D). Four weeks later, mice were sacrificed, and the tumors were harvested. The average tumor volume induced by *CHL1*-transfected cells was significantly smaller compared to tumors volume induced by non-transfected cells: *CHL1*-SUNE1 vs Vec-SUNE1 (351.92±70.69mm^3^ vs 491.56±196.38mm^3^, *P*<0.05); *CHL1*-C666 vs Vec-C666 (93.12±66.41mm^3^ vs 697.10±207.26mm^3^, *P*<0.01).

### Knockdown of *CHL1* abolishes its tumor suppressive function

To further explore the tumor-suppressive function of *CHL1*, RNAi was selected to silence/knockdown the endogenous* CHL1* expression levels in NP69 cells using siRNA. The effect of silencing was revealed by qPCR and Western blot (Fig. 2E). Functional assays were performed to characterize the effect of *CHL1* knockdown in NP69 cells. The results showed that when *CHL1* is silenced the rate of cancerous cell growth significantly increased (*P*<0.05, Student's t-test, Fig. 2F) as well as the foci formation ability (*P*<0.05, Student's t-test, Fig. 2G).

### *CHL1* arrests cell cycle by arresting at G1/S checkpoint

To explore the mechanism of the tumor suppressive function of *CHL1*, flow cytometry was utilized to make the DNA content comparison between *CHL1*- transfected and non-transfected cells. The results indicated that the ratio of cells in the S phase was significantly decreased in *CHL1*-transfected cells compared to non-transfected cells (*P*<0.05, Student's t-test, Fig. 2H), indicating that *CHL1* arrest tumor growth at the G1/S checkpoint in the growth cycle. Western blotting showed that compared to non-transfected cells, in *CHL1*-transfected cells the promoting factors cyclin D1 at the G1/S checkpoint were down-regulated while cyclin E was up-regulated (Fig. 2I).

### *CHL1* inhibits cell motility and tumor metastasis

**Figure 3:**
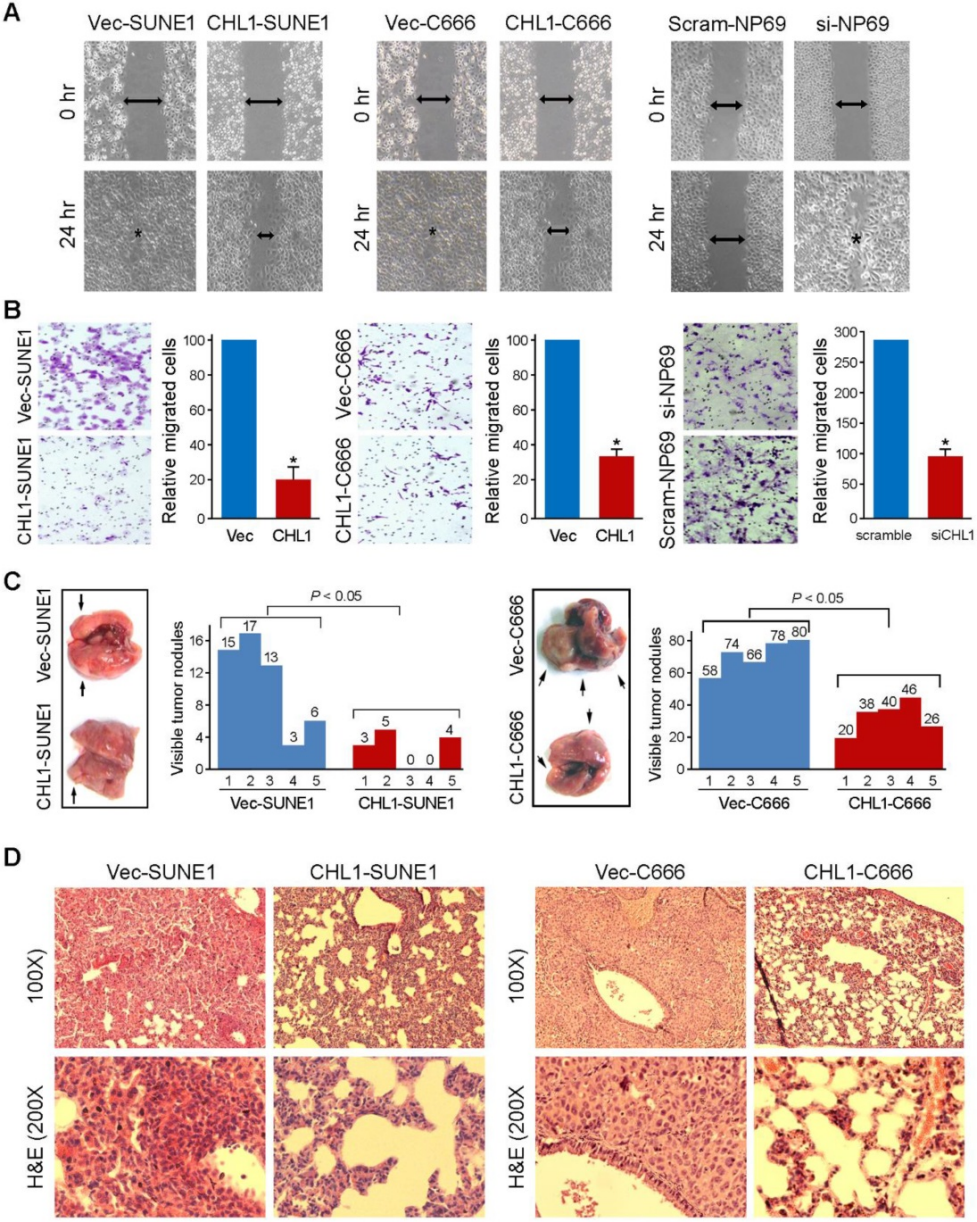
***CHL1* inhibits migration and invasion *in vitro* and in* vivo.***(A) The cell migration rate between* CHL1*-expressing cells and control cells was compared by wound-healing assay. Microscopic observation was recorded at 0 and 24 hours after scratching the cell layer. The spreading speed of *CHL1*-expressing cells along the wound edge was slower than that in control cells. (B) Representative images showed the *CHL1*-expressing and control cells that invaded through the matrigel. Number of invaded tumor cells was quantified in the right panel (*P<0.05; Student's t-test). (C) Representatives of lungs derived from SCID mice after tail vein injection of *CHL1* and vector transfected cells. The metastatic nodules at lung surface are indicated by arrows. The summary of metastatic nodules at liver surface is mean of six SCID mice for each group (* p<0.05; Student's t-test). (D) Representatives of H&E (upper) and IHC (lower) staining show lung tissue observed in SCID mice injected with vector (left) and *CHL1* (right) cells, respectively; magnification 100× (upper) and 200× (lower).

Wound-healing and cell invasion assays were used to study the effect of *CHLI* on cell motility. The wound-healing assay indicated that the ability of the cells to migrate in *CHL1*-transfected cells were significantly inhibited compared to non-transfected cells (Fig. 3A). The cell invasion assay showed that *CHL1* could significantly inhibit the invasiveness of NPC cells in *CHL1-transfected* cells, compared to the non-transfected cells (*P*<0.05, Fig. 3B). In addition, experimental metastasis assay was used to study the *in vivo* effect of *CHL1* on tumor metastasis. *CHL1- transfected and non*-transfected cells were injected into tail veins of SCID mice. The mice were sacrificed 8 weeks after to enumerate the metastatic nodules on the surface of lungs and livers. No visible metastatic nodules were observed on the surface of the liver. The appearance of nodules on the surface of the lungs significantly decreased in mice injected with *CHL1*-transfected cells compared to mice injected with non-transfected cells (*P*<0.05, independent Student's *t* test; Fig. 3C). H&E staining was performed on several sections of the lungs, and the results confirmed the nodules were metastatic tumors (Fig. 3D).

### *CHL1* inhibits epithelial-mesenchymal transitions (EMT)

**Figure 4:**
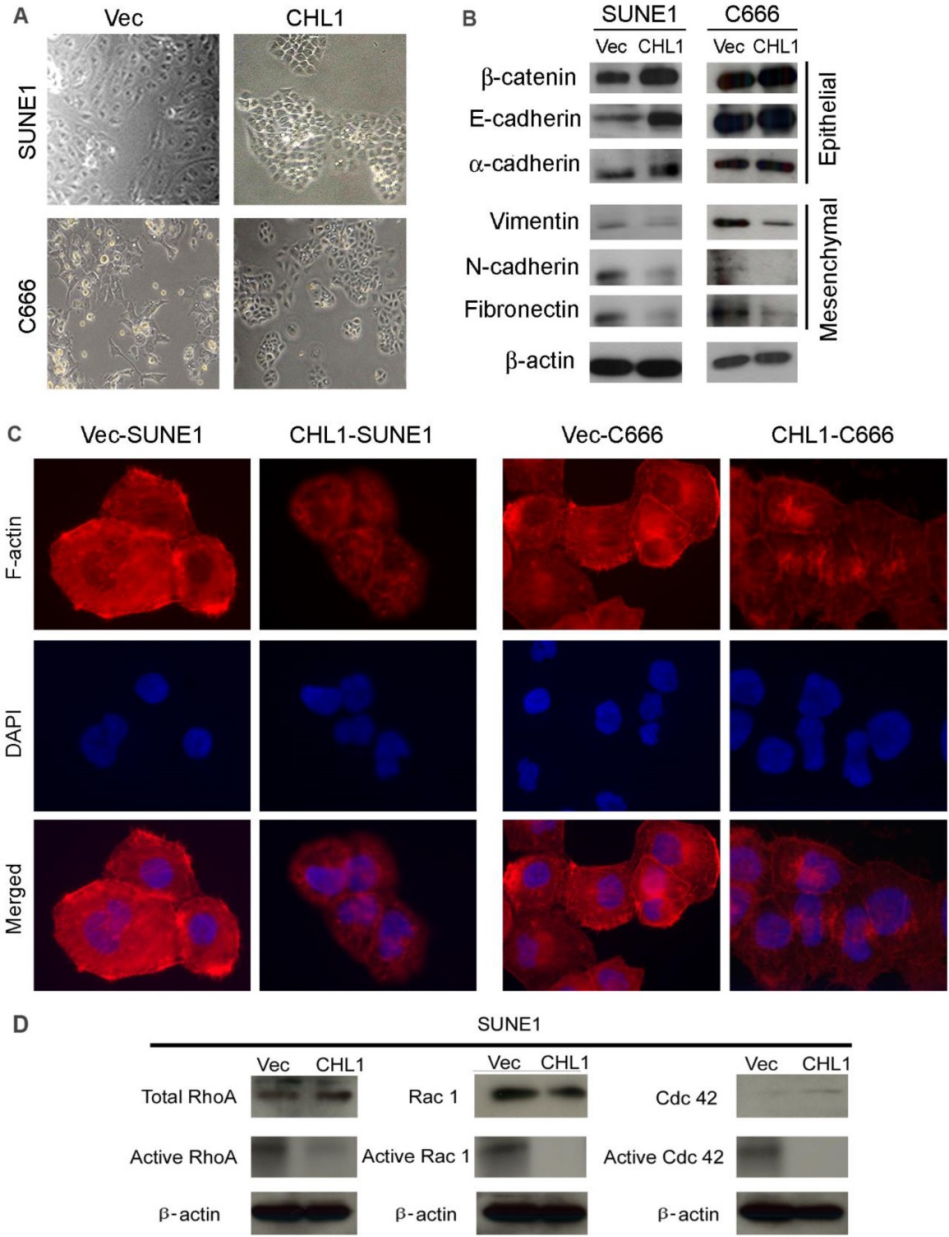
***CHL1* inhibits epithelial-mesenchymal transitions (EMT) and formation of F-actin and activation of Rho/Rac1 GTPases.** (A) Cell morphology of *CHL1*-transfected and vector-transfected cells. (B) Western blot analysis was used to compare expression levels of epithelial and mesenchymal markers between empty vector- and CHL1-transfected cells. β-actin was used as loading control. (C) Stress fiber, lamellipodia and filopodia were formed in vector-transfected cells, but not in *CHL1*-transfected cells (magnification 400×). (D) Total and active forms of Rho-GTPases, including RhoA, Rac1, and Cdc42, were compared with *CHL1*-transfected and vector-transfected cells. GTP-bound (active) forms of RhoA, Rac1, and Cdc42 were pulled down and detected by western blot. Active forms of RhoA and Rac1 were much lower in *CHL1*-transfected SUNE1 cell lines than that in vector-transfected cell lines.

Morphological analysis showed that *CHL1*-transfected cells maintained highly organized cell-cell adhesion while non-transfected cells showed spindle-shaped morphology with undermined cell-cell contact, suggesting that *CHL1* inhibit EMT (Fig. 4A). In order to test this hypothesis, Western blot analysis was performed to compare the expression of EMT markers between *CHL1*- transfected and non-transfected cells. The results showed that compared to the non-transfected cells the expression of epithelial markers in *CHL1* transfected cells increased (E-cadherin, α-cadherin and β-catenin) while its expression decreased in mesenchymal markers (fibronectin, N-cadherin and vimentin) (Fig. 4B). This result was further authenticated by IF staining (Fig. 4C), confirming that *CHL1* inhibit cell motility via the inhibition of EMT.

### *CHL1* inhibits the activation of Rho-GTPase signaling

Studies had showed that the activation of Rho-GTPase signaling pathway induces the reorganization of cytoskeletons and subsequently disrupts the cell-cell adherent junctions, the effect of *CHL1* on Rho-GTPase activity and cytoskeleton remodeling was therefore investigated. F-actin staining revealed that more stress fiber, lamellipodia and filopodia was observed in non-transfected cells, compared to *CHL1*-transfected cells (Fig. 4D), which suggests the migratory phenotype inhibited by *CHL1* may be associated with Rho-GTPases activity. To test this hypothesis, pull-down assay was used to compare the amount of RhoA, Rac1 and Cdc42 (total and GTP-bound active form) between *CHL1*- transfected and non-transfected cells. Despite having similar level of total RhoA and Rac1in *CHL1*- transfected and non-transfected cells, the pulled down active forms of RhoA-GTP and Rac1-GTP were dramatically decreased in *CHL1*-transfected cells (Fig. 4D). Also, more amount of Cdc42-GTP was pulled down in non-transfected cells although the total Cdc42 was barely detectable in both non-and *CHL1*-transfected cells (Fig. 4D).

### The interaction of *CHL1* with Integrin β1

**Figure 5:**
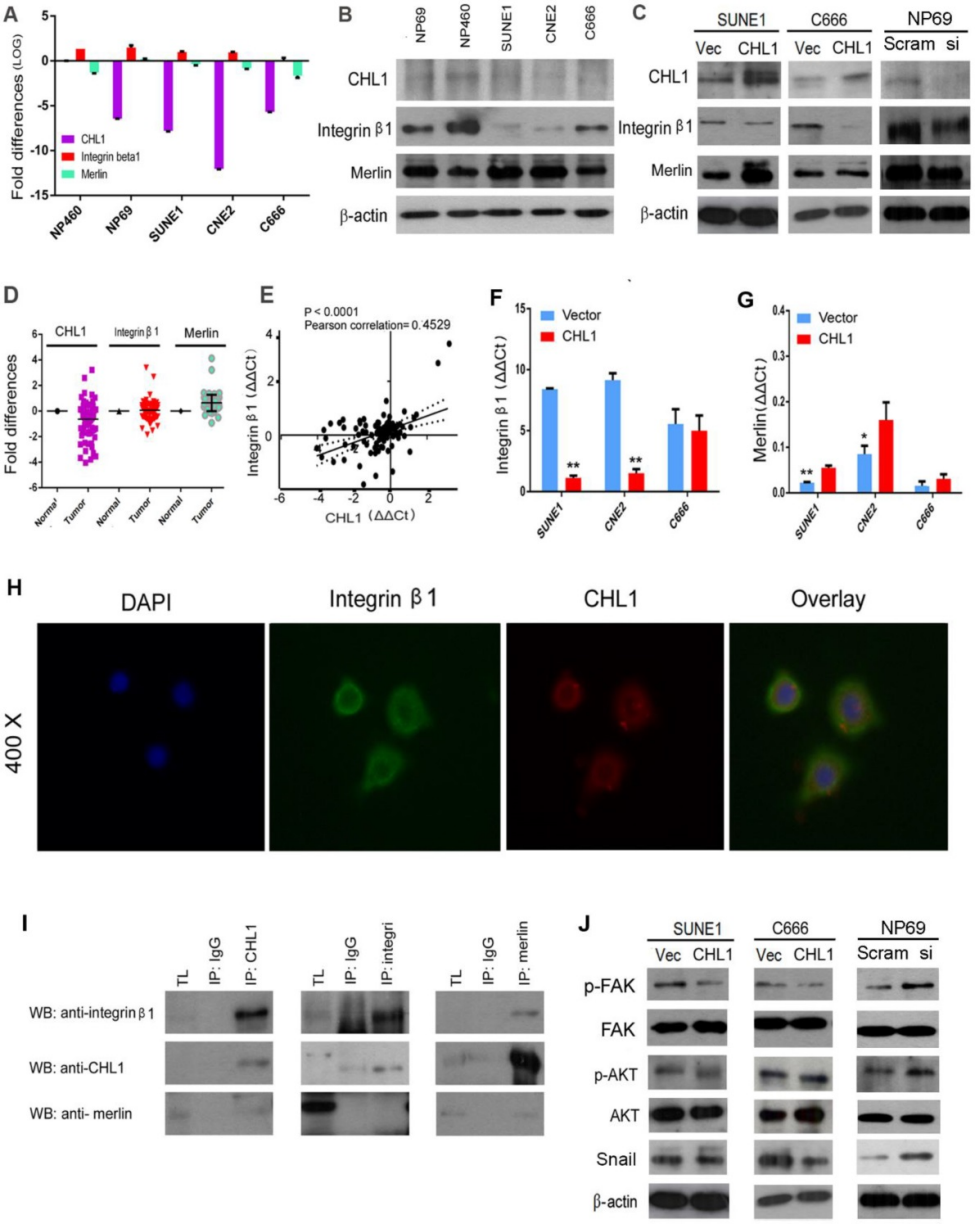
***CHL1* interacts with Integrin β1 and Merlin.** (A) qPCR analysis of *CHL1*, *Integrin β1* and *Merlin* expression in three NPC cell lines (C666, CNE2 and SUNE1), NP 460 and NP69 cell lines. *GAPDH* was set as an internal control. (B) Western blot analysis of CHL1, Integrin β1 and Merlin expression in three NPC cell lines (C666, CNE2 and SUNE1), NP 460 and NP69. (C) Expression of *CHL1, Integrin β1* and *Merlin* in *CHL1*-transfected SUNE1 and C666 was confirmed by Western blot. Empty vector-transfected cells were used as control. Expression of *CHL1, Integrin β1* and *Merlin* between scramble and si*CHL1* transfected NP69 cells was also confirmed by Western blot. (D) Expression of *CHL1, Integrin β1* and *Merlin* in non-paired primary NPC cases was compared using qPCR with its non-tumor tissue (N). (E) A positive correlation between *CHL1* and *Integrin β1* expressions in non-paired tumor tissue was determined with linear regression lines and Pearson correlation significance (p<0.0001, Pearson correlation 0.4529). (F) The expression of integrin β1 was done by qPCR between vector and *CHL1* transfected cell lines (**p<0.001, Student's t-test). (G) Merlin was done by qPCR between vector and *CHL1* transfected cell lines (*p<0.05; **p<0.001, Student's t-test). (H) Distribution of CHL1 and Integrin β1 by IF. (I)The interaction of CHL1, Integrin β1 and Merlin were performed by Co-IP. (J) The expression of p-FAK, FAK, p-AKT, AKT and Snail was done by western blot between vector and *CHL1* transfected cells, scramble and si*CHL1* transfected NP69 cells.

*CHL1* has been reported to interact with Integrin β1, with its Integrin recognition domain on the cell surface 23. Human Merlin encoded by NF2 is a magic molecule that functions as a linker to actin-cytoskeleton 24. To study the relationship of these proteins, qPCR was performed (Fig. 5A). Additionally, Western blot was also used to analyze the expression of Integrin β1 in NPC cell lines (Fig. 5B). The mRNA levels of these three proteins were investigated by qPCR using non-paired clinical NPC tissues (Fig. 5D). A significant positive correlation was observed between *CHL1* and Integrin β1 in paired and non-paired clinical tissues (Fig. 5E). Furthermore, the box plot showed that NPC tumors differed significantly from their paired tumor samples in the mean expression level of Integrin β1 (P=0.0006; Supplementary 1E & 1F). It was observed that the expressive tendency of Integrin β1 is the same with *CHL1* in the clinical samples. Kaplan-Meier analysis showed that the down-regulation of Integrin β1 and Merlin was not correlated with Disease-Free Survival (DFS) time (P >0.05) in NPC patients, but results after 30 months of observation was different, as shown in the diagram (Supplementary 1C and Supplementary 1D). Moreover, the expression of Integrin β*1* and Merlin were investigated in the non- transfected and *CHL1*transfected-cells by qPCR and western blot (Fig. 5C, Fig. 5G and Fig. 5F). To determine whether *CHL1* interacts with Integrin β1 and Merlin, two-color immunofluorescence staining was performed to visualize their subcellular distribution in NP460 (Fig. 5H). The result revealed that *CHL1* and Integrin β1 were co-localized on the cell membrane. To confirm the interaction of *CHL1* and Integrin β1, we conducted a co-immunoprecipitation (co-IP) assay. The result showed that Integrin β1 and Merlin could be pulled down by *CHL1* and vice versa, suggesting an interaction between the two proteins (Fig. 5I).

### *CHL1* inhibits Integrin β1-mediated activation of Akt signaling

To investigate how *CHL1* affect Integrin β1-mediated Akt signaling pathway, western blot was performed. Compared to vector control, our analysis revealed that overexpression of *CHL1* contributed to the reduction of phosphorylated FAK, and coincidentally decreased the downstream phosphorylation of Akt in *CHLI*-transfected cells (Ser473) (Fig. 5J). Conversely, increased phosphorylation of FAK and Akt was detected in *CHL1*-suppressed cells compared to NP69 cells (Fig. 5J). Overexpression of *CHL1* in NPC cell lines was found to decrease the expression of 'Snail', whereas knock down of *CHL1* increased its expression in NP69 cells. Collectively, the results of our findings indicated that *CHL1* exerts the tumor-suppressive function in NPCs by regulating the Akt signaling pathway.

## Discussion

In this study, a resident tumor suppressor gene *CHL1*, located on human chromosome 3p26.1, was reported to show tumor suppressive function in NPC. In this study, down-regulation of *CHL1* was frequently detected in NPCs at transcriptional level, suggesting that this gene plays a crucial role in NPC carcinogenesis. The tumor-suppressive function of *CHL1* was characterized in two NPC cell lines (SUNE1 and C666).

In all the NPC cell lines tested in which *CHL1* was not expressed or poorly expressed, frequent methylation promoters were discovered, showing that methylation is a potential mechanism for silencing *CHL1.* The expression of *CHL1* was restored after demethylation with 5'Aza. Both *in vitro* (cell growth rate and foci formation) and* in vivo* (tumor formation in nude mouse) assays showed that *CHL1* has a potent inhibitory effect on nasopharyngeal carcinoma cells.

Molecular analysis revealed that the tumor-suppressive effect of *CHL1* regulates cell cycle arrest at G1/S checkpoint by down-regulating cyclin D1, which is accumulating before S-phase and reaching its maximum. This process favors the movement of cells to the S-phase, making cyclin D1 a critical target of proliferative signals in G1. These results indicate that *CHL1* could be required at G1/S transition in NPC cells.

*CHL1* also showed strong inhibitory effects on cell motility, invasion and epithelial- mesenchymal transition. In the transition phase several of the epithelial markers were increased in the same process several mesenchymal markers were decrease in* CHL1* transfected cells compared to non-transfected cells, which indicates the negative role of *CHL1* in EMT. Also, it was revealed that *CHL1* could disrupt F-actin formation by inactivation of Rac1/Cdc42, which is an important regulator in the formation of lamellipodia and filopodia.

During tumor initiation, invasion and metastasis, the activity of Rho GTPase would affect the construction of actin cytoskeleton and the interaction of adherent junctions in cytoskeleton 25-27. The activities of RhoA, Rac and Cdc42 have been well defined in regulating the actin cytoskeleton 28. Activation of RhoA could induce formation of stress fibers, Rac1 could induce lamellipodium, while Cdc42 could induce filopodia 29. Actin cytoskeleton rearrangement promotes progression and metastasis of cancer by crossing the basement membrane and easily reaching distant organs. Therefore, we proposed that the down-regulation of *CHL1* plays a vital role in NPC invasion and metastasis. Both *in vitro* and *in vivo* assays supported the hypothesis and these findings strongly suggest that the function of *CHL1* could inhibit both cancer progression and metastasis.

Integrins are transmembrane α and β subunit comprising transmembrane receptors essential for the development and survival of organisms 30, 31. Although the role of Integrin-mediated cell-matrix adhesions in metastasis had been recognized, Integrin β1 can also act as critical receptor for a well-known oncoprotein 32. As a transmembrane glycoprotein, it could interact with *CHL1-* the integrin recognition sequence (SFT) on cell surfaces 33. As suggested by immunofluorescence assay, Integrin β1 overlaps with *CHL1* on the cell surface. The expressive tendency of Integrin β1 is the same with *CHL1*. In addition, the box plot demonstrated a significant difference in the expression level of integrin β1 between the nasopharyngeal carcinoma and non-tumor tissues, with a significant positive correlation observed between these two proteins.

Integrins activate FAK and trigger the fast recruitment of cytoskeletal proteins like talin and paxillin to its cytoplasmic domains 34. Co-localized FAK, paxillin, integrins, tailin were found in the layer of the cell surface^35^. Talin is a highly molecular cytoskeleton protein, which is mainly concentrated in the lower cell contact area 36 and at the cell-cell contacts of lymphocytes. It can directly or indirectly link integrin to the actin skeleton through interaction with NYC and alpha actin 37, 38. In addition, *in vitro* studies showed that integrin is associated with talus, although the affinity was low 39. Talin also binds to vinculin, another cytoskeletal protein associated with cell adhesion. Talin has a large C-terminal domain containing alpha helices which binds to actin and an N-terminal FERM (band 4.1,ezrin, radixin, and moesin) domain with three subdomains: F1, F2, and F3^40^-^43^. The F3 subdomain contains the Integrin binding site with the highest affinity of integrin beta tails to activate integrins sufficiently. 44.

Ezrin, Radisin, Moesin (ERM) and NF2 type neurofibromatosis (NF2) constitute a distinct subfamily of cytoskeletal members, which belong to the 4.1 band protein family 45. The ERM-NF2 protein has a conserved structure, an amino acid terminal domain, an extended alpha helix region and an actin binding carboxy terminal (C) region 46. The FERM domain pocket is the competitive site of the C- terminal self-binding region of ERM protein, and this head to tail interaction leads to "closed" monomer or in dimers. When in closed conformation the biding sites for FERM ligands and the actin is blocked, the inactive ERM is most likely confined in the cytoplasm 47, 48. During the phosphorylation of a conserved C-terminal Thr residue 49, the ERM proteins are in the open conformation, which could link plasma membrane proteins to the actin cyto skeleton^50^. While in contrast an overlapping view is observed condition when it binds to Merlin.

**Figure 6:**
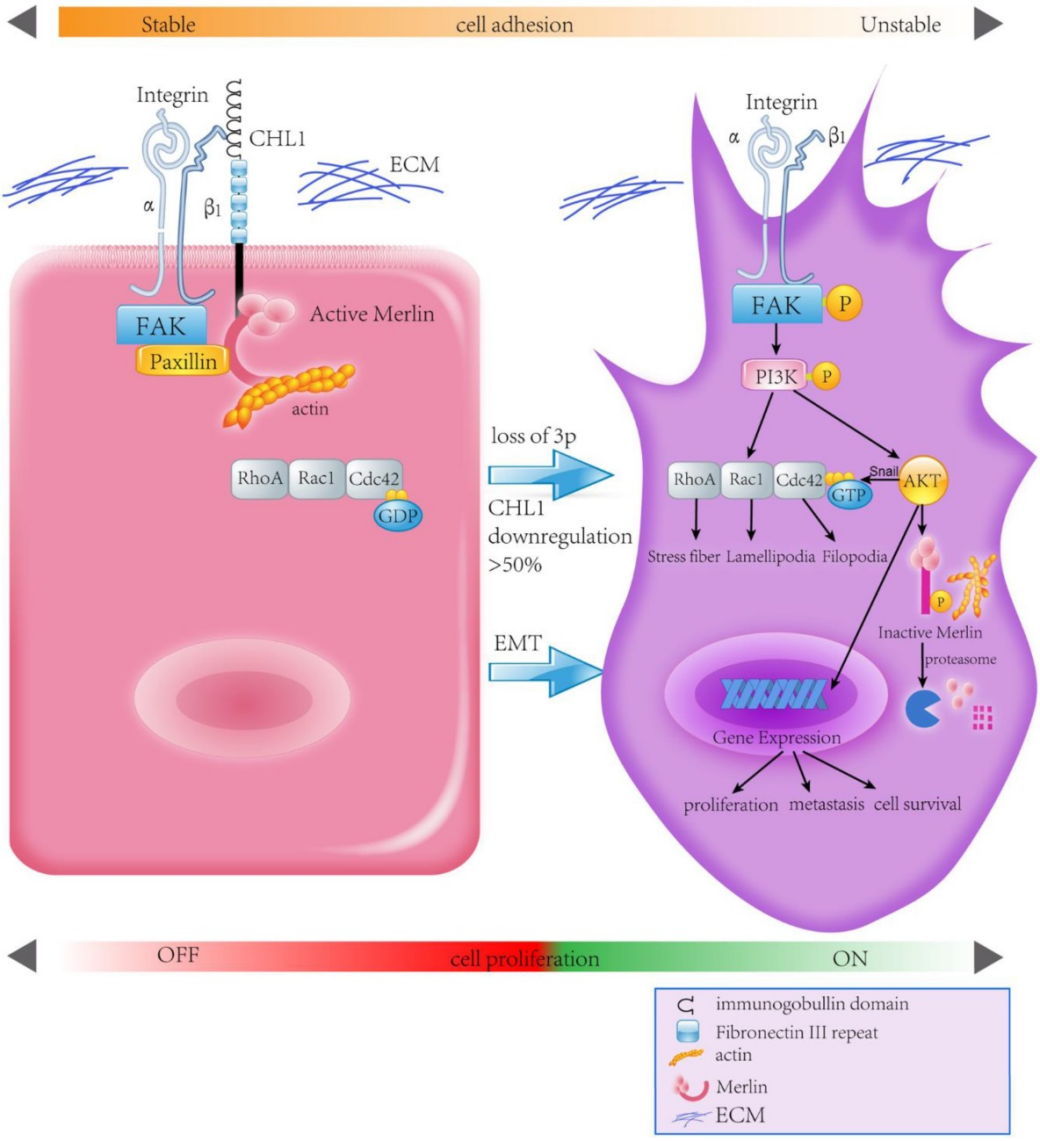
Proposed model illustrating the roles of *CHL1* during NPC carcinogenesis.

Human Merlin encoded by NF2 is a magic molecule that functions as a linker to actin-cytoskeleton 24 and as a tumor suppressor in many brain cancers, including schwannomas 51, meningioma^52^-^57^, and ependymomas 51, 58. By using the pull-down assay the relationship between *CHL1*, Integrin-β1, and Merlin has been demonstrated. When *CHL1* is lost, integrin beta1-AKT pathway would be activated by phosphorylation and inactivation of Merlin, so that *CHL1* it no longer plays its tumor suppressive role (Fig. 6).

In conclusion, this study reveals *CHL1* as a tumor suppressor gene for both nasopharyngeal carcinoma progression and metastasis. *CHL1* plays this role through a mechanism involving the interaction of Integrin β1 and Merlin, and subsequently the inactivation of a host of downstream signaling cascades, called the PI3K/Akt pathway. In view of the important role of Akt and Merlin in the development of tumor, a more comprehensive understanding of the mechanism of *CHL1* in nasopharyngeal carcinoma will provide a more effective therapeutic strategy for the treatment of nasopharyngeal cancer patients.

## Supplementary Material

Supplementary figures and tables.
