# *CHL1* suppresses tumor growth and metastasis in nasopharyngeal carcinoma by repressing PI3K/AKT signaling pathway via interaction with Integrin β1 and Merlin: Erratum

**DOI:** 10.7150/ijbs.79278

**Published:** 2023-01-05

**Authors:** Juan Chen, Chen Jiang, Li Fu, Cai-Lei Zhu, Yan-Qun Xiang, Ling-Xi Jiang, Qian Chen, Wai Man Liu, Jin-Na Chen, Li-Yi Zhang, Ming Liu, Chao Chen, Hong Tang, Bo Wang, Sai Wah Tsao, Dora Lai-Wan Kwong, Xin-Yuan Guan

**Affiliations:** 1 Departments of Clinical Oncology, Li Ka Shing Faculty of Medicine, The University of Hong Kong, Hong Kong, China;; 2 Department of Clinical Oncology, The Seventh Affiliated Hospital, Sun Yat-sen University.; 3 Departments of Pathology, Li Ka Shing Faculty of Medicine, The University of Hong Kong, Hong Kong, China;; 4 Departments of Anatomy, Li Ka Shing Faculty of Medicine, The University of Hong Kong, Hong Kong, China;; 5 State Key Laboratory of Oncology in Southern China, Sun Yat-Sen University Cancer Center, Guangzhou, China;; 6 Department of Nasopharyngeal, Sun Yat-Sen Cancer Center, Guangzhou, China.; 7 Guangdong Key Laboratory for Genome Stability & Disease Prevention, Department of Pharmacology and Shenzhen University International Cancer Research Centre, Shenzhen University school of Medicine, Shenzhen, China.; 8 Department of Orthopedics, Union Hospital, Tongji Medical College, Science and Technology of Huazhong University, Wuhan, China.

In our paper, two representative images were misplaced into the manuscript during the stage of figure preparation. Specifically, one wound-healing image (CHL1-C666 / 0hr) in Figure 3A and the western blotting of Fibronectin of SUNE1 in Figure 4B were incorrect. Our replacement data came from the original data (Original Figure). Corrected figures are provided below. This correction does not alter the interpretation of the results and conclusion.

We apologize for our carelessness in preparing figures and for any inconvenience caused.

## Figures and Tables

**Figure 1:**
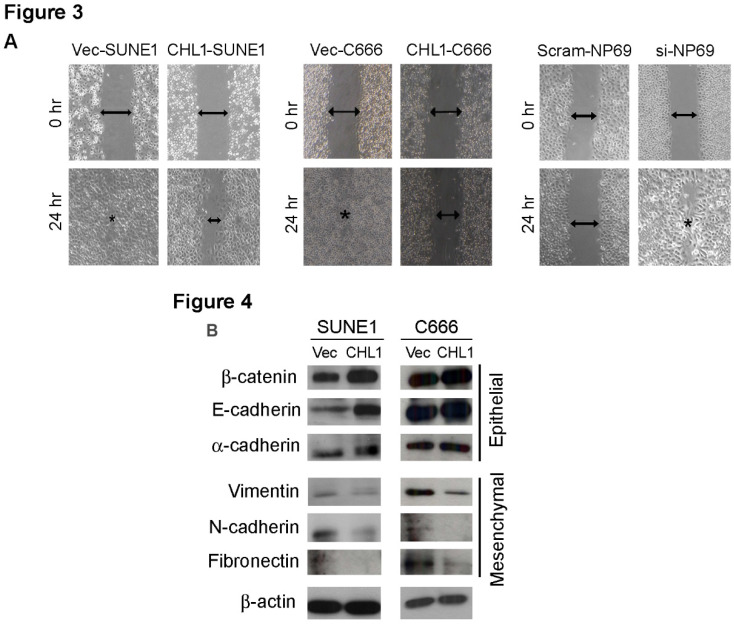
Correct Figure 3A and Figure 4B.

