# Supplementary material for: CHL1 suppresses tumor growth and metastasis in nasopharyngeal carcinoma by repressing PI3K/AKT signaling pathway via interaction with Integrin β1 and Merlin
Source: Int J Biol Sci. 2019 Jul 11;15(9):1802–15. doi: 10.7150/ijbs.34785 (PMC6743306; doi:10.7150/ijbs.34785)
Supplement: Supplementary file 1 — Supplementary figures and tables. [file ijbsv15p1802s1.pdf]

Supplementary Table 1. Association between *CHL1*, *Merlin*, and *Integrin β1* down-regulation and clinic-pathologic characteristics of 95 NPC cases.

		Integrin β1		P	Merlin		P	CHL1		P
		0	1		0	1		0	1	
AGE	<45	24	22	1.0	19	27	0.148	22	24	0.837
	>=45	25	23		28	20		25	23	
SEX	M	35	36	0.471	35	36	1	34	37	0.632
	F	13	9		11	11		12	10	
T	T1-2	3	4	0.706	4	3	1	3	4	1
	T3-4	46	41		43	44		44	43	
N	N0-1	34	26	0.286	31	29	0.830	29	31	0.830
	N2-3	15	19		16	18		18	16	
DISEASE-SPECIFIC	NO	41	41	0.361	40	42	0.759	40	42	0.759
	YES	8	4		7	5		7	5	
METASTASIS	NO	46	43	1	45	44	1	46	43	0.361
	YES	3	2		2	3		1	4	
RELAPSE	NO	43	42	0.490	41	44	0.486	41	44	0.486
	YES	6	3		6	3		6	3	

Supplementary Table 2. Antibodies used in this study

CHL1	HPA00345	sigma
GFP	sc-9996	Santa Cruz
Integrin β1	EP1041Y	Novus
Cyclin D1	2978	Cell Signaling
α-catenin	3236	Cell Signaling
β-catenin	9562	Cell Signaling
E-cadherin	3195	Cell Signaling
Snail	3895	Cell Signaling
AKT	9272	Cell Signaling
p-AKT	9271	Cell Signaling
CDK4	ab95255	Abcam
β-actin	ab 8227	Abcam
CDK2	14-475	Upstate Biotechnology
Cyclin E	A00543-1	Boster
Vimentin	YM 3158	ImmunoWay
Fibronectin	YM3137	ImmunoWay
Snail	3895S	Cell Signaling

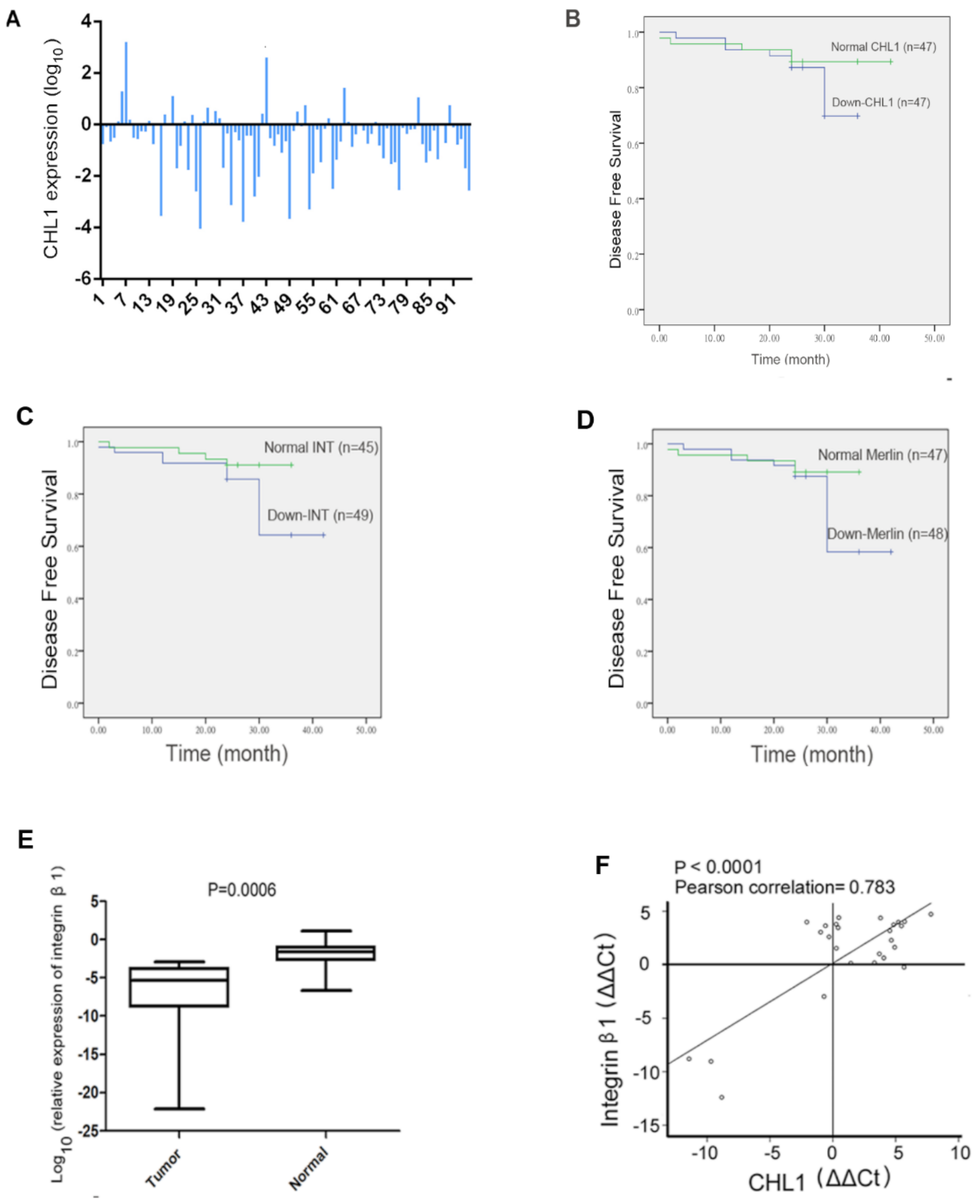

Chen et al, suplliment Fig 1

Supplementary Figure (A) Expression of *CHL1* in 95 primary NPC cases was compared using qPCR with one mixed normal tissue (N). *GAPDH* was set as an internal control. (B) Kaplan–Meier analysis was used analyze the down-regulation of *CHL1* associated with the Disease-Free Survival (DFS) time (P >0.05). (C)

Kaplan–Meier analysis was used analyze the down-regulation of *Integrin β1* associated with the Disease-Free Survival (DFS) time (P >0.05). (D) Kaplan–Meier analysis was used analyze the down-regulation of *Merlin* associated with the Disease-Free Survival (DFS) time (P >0.05). (E) (A) Fold change of *Integrin β1* in 15 primary NPC tissues and their adjacent non-tumor tissues (N) were detected using qRT-PCR. *Integrin β1* was normalized by internal control GAPDH (**P*<0.01, independent Student *t* test). (F) A positive correlation between *CHL1* and *Integrin β1* expressions in paired tumor tissue was determined with linear regression lines and Pearson correlation significance (p<0.0001, Pearson correlation 0.783).
